# Silencing of *GhKEA4* and *GhKEA12* Revealed Their Potential Functions Under Salt and Potassium Stresses in Upland Cotton

**DOI:** 10.3389/fpls.2021.789775

**Published:** 2021-12-07

**Authors:** Yi Li, Zhen Feng, Hengling Wei, Shuaishuai Cheng, Pengbo Hao, Shuxun Yu, Hantao Wang

**Affiliations:** ^1^State Key Laboratory of Cotton Biology, Institute of Cotton Research of Chinese Academy of Agricultural Sciences, Anyang, China; ^2^Zhengzhou Research Base, State Key Laboratory of Cotton Biology, Zhengzhou University, Zhengzhou, China

**Keywords:** upland cotton, K^+^ efflux antiporter (KEA), salt and potassium stresses, virus-induced gene silencing, K^+^ transport

## Abstract

The K^+^ efflux antiporter (KEA) mediates intracellular K^+^ and H^+^ homeostasis to improve salt tolerance in plants. However, the knowledge of KEA gene family in cotton is largely absent. In the present study, 8, 8, 15, and 16 putative KEA genes were identified in *Gossypium arboreum*, *G. raimondii*, *G. hirsutum*, and *G. barbadense*, respectively. These KEA genes were classified into three subfamilies, and members from the same subfamilies showed similar motif compositions and gene structure characteristics. Some hormone response elements and stress response elements were identified in the upstream 2000 bp sequence of *GhKEAs*. Transcriptome data showed that most of the *GhKEAs* were highly expressed in roots and stems. The quantificational real-time polymerase chain reaction (qRT-PCR) results showed that most of the *GhKEAs* responded to low potassium, salt and drought stresses. Virus-induced gene silencing (VIGS) experiments demonstrated that under salt stress, after silencing genes *GhKEA4* and *GhKEA12*, the chlorophyll content, proline content, soluble sugar content, peroxidase (POD) activity and catalase (CAT) activity were significantly decreased, and the Na^+^/K^+^ ratio was extremely significantly increased in leaves, leading to greater salt sensitivity. Under high potassium stress, cotton plants silenced for the *GhKEA4* could still maintain a more stable Na^+^ and K^+^ balance, and the activity of transporting potassium ions from roots into leaves was reduced silenced for *GhKEA12*. Under low potassium stress, silencing the *GhKEA4* increased the activity of transporting potassium ions to shoots, and silencing the *GhKEA12* increased the ability of absorbing potassium ions, but accumulated more Na^+^ in leaves. These results provided a basis for further studies on the biological roles of KEA genes in cotton development and adaptation to stress conditions.

## Introduction

With changes in the global environment, crops are facing abiotic stress environments such as soil salinization, drought and extreme temperatures in the process of production. Salt stress is one of the most important abiotic stress factors, that seriously affect the growth, development and survival of plants (Munns, 2002; Liu et al., 2010). Although cotton is considered to be a salt-tolerant and drought-tolerant crop, the restrictions on cotton growth and yield caused by high salt and drought stress cannot be ignored. Research has shown that when the salt concentration exceeds a certain threshold, the normal physiological function and material metabolism of cotton are significantly affected, and the growth and development of cotton are inhibited, which leads to a decrease in yield and a deterioration in fiber quality (Sharif et al., 2019). On the one hand, when the salt concentration is high, the content of sodium ions is significantly higher than the content of potassium ions, resulting in a higher Na^+^/K^+^ ratio, which destroys the water balance in the plant cells (Hauser and Horie, 2010); on the other hand, salt stress can lead to plant membrane damage, enzyme activity inhibition, metabolic disorders, etc., resulting in plant growth inhibition and even death (Munns and Tester, 2008). K^+^ is one of a large number of mineral elements needed by plants, and it also plays an important role in plant salt tolerance (Britto and Kronzucker, 2008). Plants balance excessive Na^+^ by accumulating K^+^ to reduce the damage caused by salt stress (Cuin et al., 2003). Therefore, it is very important for plants to absorb and transport potassium ions effectively.

In plants, there are many proteins that consume energy to absorb potassium ions from the outside environment, that is, proteins that carry out active transport. These proteins, called potassium transporters, are divided into three families according to their structures and functions: the KUP/HAK/KT family, the HKT family and the CPA family (Gierth and Maser, 2007). The CPA (Cation Proton Antiporter) family mediates the homeostasis of ions and pH in cells, maintains osmotic balance, and regulates plant growth and development and signal transduction. The CPA family is divided into two subfamilies: CPA1 and CPA2. The CPA1 subfamily is mainly NHX (Na^+^/H^+^ exchanger) transporters, and the CPA2 subfamily includes KEA (K^+^ efflux antiporter) and CHX (Cation/H^+^ exchanger) transporters (Chanroj et al., 2011). The numbers of CHX gene families have increased dramatically from charophyte algae to flowering plants (Aranda-Sicilia et al., 2012). Fan et al. (2020) studied the molecular evolution and expansion of KUP gene family in *Gossypium hirsutum* and *G. barbadense*, and found that KUP family genes showed different expression levels under various stress treatment. In our previous study, KUP/HAK/KT gene family and NHX gene family have been identified and found to be involved in abiotic stress response (Fu et al., 2020; Yang et al., 2021). Compared with the diverse CHX gene family, the number of KEA family members varies from plant to plant. Seven, 4, 4, 12, 6, and 24 KEA family members were identified in *Populus trichocarpa*, *Oryza sativa*, *Sorghum bicolor*, *Glycine max*, *Zea mays*, and *Triticum aestivum*, respectively (Ye et al., 2013; Sze and Chanroj, 2018; Sharma et al., 2020). The KEA family in higher plant was first reported in *Arabidopsis thaliana* and originated from bacterial glutathione-regulated K^+^ efflux antiporters KefB and KefC with an N-terminal Na^+^/H^+^ exchanger domain and a C-terminal KTN-NAD (H)-binding domain (also known as the TrkA-N domain) (Maser et al., 2001; Choe, 2002; Chanroj et al., 2012). The AtKEA family was divided into KEAI and KEAII. KEAI was divided into Ia and Ib according to its N-terminal domain and had a complete C-terminal KTN domain, which was closely related to EcKefB and EcKefC proteins (Booth, 2003; Fujisawa et al., 2007; Chanroj et al., 2012). KEAII lost the KTN domain at the C-terminus and had high homology with cyanobacteria, which was similar to the transmembrane coiled coil protein 3 (TMCO3) (Chanroj et al., 2012). The first research on the KEA family showed that *AtKEA2* was located on the chloroplast, and was involved in the regulation of K^+^ and the pH of the plastid (Aranda-Sicilia et al., 2012). The inner envelope *AtKEA1* and thylakoid *AtKEA3* transporters were reported to be involved in chloroplast function, osmotic regulation, photosynthesis and pH regulation, and resisted high potassium and high hygromycin in yeast (Zheng et al., 2013; Kunz et al., 2014). In addition, genetic analysis showed that *AtKEA4*, *AtKEA5*, and *AtKEA6* had similar tissue expression patterns, and they cooperated with endosomes *NHX5* and *NHX6* to promote the pH homeostasis and salt tolerance (Zhu et al., 2018). Therefore, the KEA gene family not only plays a significant role in K^+^ transport but also may be involved in abiotic stress responses in plants.

However, to date, there have been no reports on the genome-wide identification and characterization of the cotton KEA gene family members. With the publication of the cotton genome sequence and transcriptome data, it is possible to comprehensively identify and analyze the KEA gene family, which is also a key step in studying the function of cotton KEA genes. In this study, members of the KEA gene family in *G. arboreum*, *G. raimondii*, *G. hirsutum*, and *G. barbadense* were identified. The physical and chemical properties, chromosome distributions, gene structures, evolutionary relationships, gene replications and expression patterns were comprehensively analyzed. The functions of *GhKEA4* and *GhKEA12* under salt and potassium stresses were preliminarily explored by virus-induced gene silencing (VIGS) experiments. This research provides basic data for further study on the function of KEA genes in cotton.

## Materials and Methods

### Identification of the K^+^ Efflux Antiporter Gene Family

The genome databases of *Gossypium arboreum* (accession number: PRJNA382310) (Du et al., 2018), *Gossypium raimondii* (accession number: PRJNA171262) (Paterson et al., 2012), *Gossypium hirsutum* (accession number: PRJNA433615) (Wang M. et al., 2019) and *Gossypium barbadense* (accession number: PRJNA433615) (Wang M. et al., 2019) were obtained from the CottonGen database^1^ (Yu et al., 2014). In addition, the genome databases of *Arabidopsis thaliana* (accession number: PRJNA10719), *Oryza sativa* (accession number: PRJNA448171), *Zea mays* (Schnable et al., 2009), *Populus trichocarpa* (Tuskan et al., 2006), *Sorghum bicolor* (accession number: PRJNA374837), *Triticum aestivum* (Mayer et al., 2014) and *Glycine max* (accession number: ACUP00000000) were downloaded from the phytozome database^2^. The protein sequences of AtKEA1-AtKEA6 were used to construct the hidden markov model (HMM) of the conserved domain of a specific KEA gene family. The HMMER 3.0 program and the constructed HMM model were used to search the predicted KEAs from the above plant genomes. The default parameter of the e-value threshold was set at 1e-50. Then, the NCBI Conserved Domain Database^3^ and SMART database^4^ were used to confirm whether the candidate protein sequences contain the special domain of the KEA family (Letunic et al., 2015). The protein sequence length, molecular weight (Mw), isoelectric point (pI), grand average of hydropathicity (GRAVY) and subcellular localization of the identified KEA members were predicted from the ExPasy website^5^ and ProtComp 9.0^6^ (Artimo et al., 2012).

### Sequence Alignments and Phylogenetic Analysis

All the identified KEA protein sequences from *G. arboreum*, *G. raimondii*, *G. hirsutum*, *G. barbadense*, *Arabidopsis thaliana*, *Oryza sativa*, *Zea mays*, *Populus trichocarpa*, *Sorghum bicolor*, *Triticum aestivum*, and *Glycine max* were aligned by ClustalX 2.0 (Larkin et al., 2007). The phylogenetic tree was constructed using the neighbor-joining (NJ) method of MEGA 7.0 with the p-distance model and 1000 bootstrap replications (Kumar et al., 2016).

### Locations of K^+^ Efflux Antiporter Gene on Cotton Chromosomes and Gene Duplication Analysis

The chromosome physical locations of the KEA gene family were extracted from the genome annotation file information of *G. hirsutum*, *G. raimondii*, *G. barbadense*, and *G. arboreum*, and the positions of KEA genes on chromosomes were visualized with Map Chart 2.2 software (Voorrips, 2002). The replication gene pairs of *G. hirsutum*, *G. raimondii*, and *G. arboreum* were detected by MCScanX software (Wang et al., 2012), and gene replication was confirmed according to the following conditions: the coverage of the alignment sequence was ≥80% of the longer gene; the similarity of the regions on the alignment was ≥80%; tightly linked genes on the same chromosome were considered tandem duplications. Circos was adopted to plot the diagram of segmental duplication events on chromosomes (Krzywinski et al., 2009). KaKs_Calculator 2.0 software was used to calculate the non-synonymous mutation rate (Ka) and synonymous mutation rate (Ks) of KEA gene replication (Wang et al., 2010).

### Gene Structure and Conserved Motif Analysis

The GhKEA proteins were used for multiple sequence alignment by ClustalX 2.0. The exon-intron structures of upland cotton KEA genes were analyzed on the Gene Structure Display Server (GSDS 2.0^7^) (Hu et al., 2015). The conserved motifs of KEA proteins were identified by the MEME program. The optimization parameters were set as follows: size distribution, zero or once per sequence; motif count, 10; pattern width, between 6 and 50 residues (Bailey et al., 2006).

### Promoter Region *Cis*-Acting Element Analysis

DNA sequences 2000 bp upstream of the KEA start codon (ATG) were retrieved from the *G. hirsutum* genome database and submitted to PlantCARE^8^ for analysis of *cis*-acting elements (Lescot et al., 2002).

### Gene Expression Pattern Analysis

The raw RNA-sequencing data of *G. hirsutum* TM-1 in different tissues were obtained from previously reported transcriptome data (accession number: PRJNA248163) (Zhang et al., 2015). TBtools was used to draw a heatmap, using row-scale and zero to one scale methods, showing the expression patterns of *GhKEAs* (Chen et al., 2020).

### Plant Materials and Treatments

In this study, *G. hirsutum* Texas Marker-1 (TM-1) was cultivated by hydroponics with hoagland nutrient solution by Solarbio Biology Co., Ltd., and grown in a climate-controlled chamber with a light/dark cycle of 16 h at 28°C/8 h at 22°C. When the third true leaf was unfolded (approximately 4 weeks), it was treated with NaCl (300 mMol/L), KCl (0.03 mMol/L), PEG6000 (30%) and control group. Samples were taken at 0, 1, 3, 6, 12, and 24 h respectively. All the samples were immediately frozen in liquid nitrogen and stored at –80°C.

### Construction of the Virus-Induced Gene Silencing Vector and Determination of Physiological Parameters

The *GhKEA4* (*Ghir_D12G011600.1*) and *GhKEA12* (*Ghir_A06G009360.1*) fragments of 300 nt were introduced by primers, respectively. The fragments of the above genes were then ligated into the pYL156 vector. The primers used for vector construction are listed in Table 1. The recombinant vector was transformed into *Agrobacterium tumefaciens* LBA4404. According to the method mentioned by Gao et al. (2016), we injected LBA4404 bacterial solution carrying pYL156 (empty vector), pYL156-GhKEA4, pYL156-GhKEA12, pYL156-CLA1 (positive control) and pYL192 (helper vector) into the cotyledons of TM-1. After 24 h of dark treatment, the cotton plants were moved to a greenhouse with 12 h of light/12 h of darkness for approximately 15 days, and then treated with NaCl. Before salt treatment, 5–6 leaves of control plants and VIGS plants were taken and weighed immediately. Then the leaves were placed in a petri dish with filter paper, placed in a 28°C incubator, set to three repeats, and weighed every other hour. Water loss rate of isolated leaves = (leaf fresh weight-leaf dry weight)/leaf fresh weight × 100%. The experiment was repeated three times independently.

**TABLE 1:** List of the primers used to construct PYL-156 vectors in present study.

ID	Name	Forward primer	Reverse primer
Ghir_D12G011600.1	GhKEA4-156	AAGGTTACCGAATTCTCTAGAATCAAATTTTCCTGTCATTGC	GAGCTCGGTACCGGATCCACATCGTGCAGCTCAAAA
Ghir_A06G009360.1	GhKEA12-156	AAGGTTACCGAATTCTCTAGAATTTGCTTGTGCTGGACAAC	GAGCTCGGTACCGGATCCAGAAATACACCAACAAATACACC

After 24 h of 300 mM NaCl treatment, 0.1 g of sample powder mixed by at least 20 cotton plants were taken to determine the total chlorophyll content, soluble sugar content and proline (Pro) content, as well as catalase (CAT) activity and peroxidase activity (POD). And three biological replicates were performed. Chlorophyll was extracted with a ratio of ethanol to acetone (1:1), and the absorbance was measured at 663 and 645 nm (Sun et al., 2013). Other indicators used the kit developed by Solarbio Biology Co., Ltd., and the specific operation steps were guided according to the operating instructions.

### Measurement of K^+^ and Na^+^ Concentration

First, control plants and plants silenced for the target gene were sampled before and after treatment with 300 mM NaCl (high salt), 0.2 mM KCl (low potassium) and 10 mM KCl (high potassium). The samples were then quickly put at 105°C for 30 min to kill and dried at 80°C for 48 h until the weight was unchanged. 0.5 g of plant sample was weighed and used for determination of Na^+^ and K^+^ contents (Bao, 2005).

### RNA Extraction and Quantificational Real-Time Polymerase Chain Reaction Analysis

Total RNA was isolated from the collected samples using the RNA-prep Pure Plant Kit (TIANGEN, Beijing, China). One microgram of RNA was reverse transcribed into cDNA using the Prime Script RT Reagent kit (Takara, Japan), and the system was diluted fivefold upon completion of reverse transcription for the next experiment. SYBR Premix Ex Taq (Takara) and the ABI 7500 Real-time PCR system (Applied Biosystems) were used to carry out quantificational real-time polymerase chain reaction (qRT-PCR) experiments. The protocol was performed as follows: step 1: 95°C for 30 s; step 2: 40 cycles of 95°C for 5 s, and 60°C for 34 s; and step 3: melting curve analysis. For each sample, three biological repeats were performed to obtain reliable results (Livak and Schmittgen, 2001). The specificity of the qRT-PCR primers was demonstrated by ePCR and melting curves. The cotton histone-3 gene (GenBank accession number AF024716) was used as an internal reference gene to normalize the expression level of the target gene (Liu et al., 2017). The data were calculated according to the 2^–ΔΔCt^ formula (Livak and Schmittgen, 2001). Gene specific primers for qRT-PCR were designed by Oligo 7 (Table 2).

**TABLE 2:** List of the primers used for quantitative real-time PCR in present study.

ID	Name	Forward primer	Reverse primer
Ghir_A08G013810.1	GhKEA1	CGTGCACTGGACCTTCCTGTTT	CTGCAGCACATGCTCTTTCAGC
Ghir_A12G011370.1	GhKEA2	GGGGATTTCAATGGCCCTCACA	ATCAAGGGCAACGAATGG
Ghir_D08G014670.1	GhKEA3	TAGAAAAGGCTGGTGCTACGGC	CGTTGATCGTTGCCGCAATCTC
Ghir_D12G011600.1	GhKEA4	TTTAGGTCTCGCCATCTTGCGG	TTATCGTCCGAAGAATCCGGCG
Ghir_D02G024430.1	GhKEA5	AGGGCTGCTGATTTCGTTGACA	CAGAAGCAAGCGGAGTCGACAA
Ghir_A03G023000.1	GhKEA6	AAGCCGGTGCAACAGATGCAAT	AGCCTTATCGATTCTCGCCTGC
Ghir_D13G021510.1	GhKEA7	ATCGAGCAGATGATGCACCGAC	AAAAGCAATGCCACCGCATGTT
Ghir_A13G020690.1	GhKEA8	TTTGCTGCTCTTTTCCTCGCCA	GTTGTGCCCAGAAGTAGCAGGT
Ghir_A07G004510.1	GhKEA9	GCGAAGGGAGCATTGCCAAAAT	GCTACAGTCTCCAGTACAGCTTGC
Ghir_D06G009670.1	GhKEA10	CAACATGCTTCACGGCCAAGTC	AGGAAGGCCTGTACGGGACAAT
Ghir_A08G025770.1	GhKEA11	TCTTAGTTTGGTGACTACT	GAATGGCAATGCGGCATC
Ghir_A06G009360.1	GhKEA12	ACCGAAGGACGGTACTTTTGCC	GTCTTCACTCTGGCCACGGTTT
Ghir_D08G026630.1	GhKEA13	GATGCTCATTTTCCGGGT	TTAAGAAATTGGCAGTAAAAGT
Ghir_D07G004540.1	GhKEA14	TGGCGATTTGCTCCTCGAGA	GCCGGTACCCGTCACCGA
Ghir_A07G015190.1	GhKEA15	AGGTTAAGTTCCATGAAG	ACTTAGTTACCTGCAGGA

## Results

### Identification and Characteristics of K^+^ Efflux Antiporters in *Gossypium* spp.

Based on the HMM model of the KEA specific protein conserved domain constructed by the ATKEA1–ATKEA6 protein sequences, a total of 8, 8, 15, and 16 KEA members were identified from *G. arboreum*, *G. raimondii*, *G. hirsutum*, and *G. barbadense*, respectively (Table 3). All these putative genes were detected to contain the typical Na^+^/H^+^ exchanger domain (pfam: PF00999) and some members contained the TrkA-N domain (pfam: PF02254) of the KEA gene family in CDD and SMART databases. The confirmed members of the KEA gene family were named *GaKEA1* to *GaKEA8, GrKEA1* to *GrKEA8*, *GhKEA1* to *GhKEA15* and *GbKEA1* to *GbKEA16* according to the size of the e-values screened. These putative GaKEAs encoded proteins ranging from 595 amino acids (aa) (GaKEA8) to 1209 aa (GaKEA2), while GrKEAs encoded ranging from 573 aa (GrKEA6) to 1209 aa (GrKEA2), GhKEAs encoded 121 aa (GhKEA15) to 1210 aa (GhKEA2) and GbKEAs encoded 441 aa (GbKEA16) to 1209 aa (GbKEA2 and GbKEA4). Most members of the KEA family have transmembrane domains, which means that these proteins may be located on the membrane. The location of these proteins on chromosomes and the predicted molecular weight (MW), isoelectric point (pI) and grand average of hydropathicity (GRAVY) are shown in Table 3.

**TABLE 3:** Basic information for KEAs in *Arabidopsis thaliana*, *G. arboreum*, *G. raimondii*, *G. hirsutum*, and *G. barbadense*.

ID	NAME	Chr.	Length	Mw(kDa)	pI	GRAVY	Subcellular localization	Location
AT1G01790	ATKEA1	1	1193	128.034	5.22	0.08	Chloroplast	284350–291203
AT4G00630	ATKEA2	4	1185	127.605	5.11	0.086	Chloroplast	261246–268097
AT4G04850	ATKEA3	4	776	83.7907	5.53	0.369	Chloroplast	2452664–2457767
AT2G19600	ATKEA4	2	592	64.2494	5.91	0.589	Plasma membrane	8478970–8483854
AT5G51710	ATKEA5	5	568	61.5984	5.84	0.613	Plasma membrane	21004251–21008849
AT5G11800	ATKEA6	5	597	64.3917	7.1	0.599	Plasma membrane	3803315–3808273
Ga08G1558.1	GaKEA1	8	1205	130.33	5.37	0.03	Chloroplast	104013215–104021677(–)
Ga12G1877.1	GaKEA2	12	1209	129.982	5.23	0.12	Chloroplast	30757828-30767684(+)
Ga03G2727.1	GaKEA3	3	972	106.397	8.58	0.199	Chloroplast	135169648–135176726(+)
Ga07G1590.1	GaKEA4	7	867	94.8015	5.99	–0.089	Chloroplast	32599799–32639902(+)
Ga13G2411.1	GaKEA5	13	599	64.6596	6.03	0.557	Plasma membrane	119381339–119388157(+)
Ga07G0481.1	GaKEA6	7	1119	122.991	6.28	0.24	Plasma membrane	5200681–5212745(–)
Ga08G2879.1	GaKEA7	8	968	104.966	8.39	0.113	Plasma membrane	128629936–128641870(–)
Ga06G0996.1	GaKEA8	6	595	64.6759	5.57	0.559	Plasma membrane	32202261–32208138(–)
Gbar_A08G014340.1	GbKEA1	A08	1180	127.738	5.39	0	Chloroplast	96262469–96271708(–)
Gbar_A12G011370.1	GbKEA2	A12	1209	130.115	5.26	0.117	Chloroplast	73274768–73284631(–)
Gbar_D08G015210.1	GbKEA3	D08	1205	130.231	5.44	0.057	Chloroplast	47645632–47654679(+)
Gbar_D12G011550.1	GbKEA4	D12	1209	129.909	5.26	0.092	Chloroplast	36785162–36794816(–)
Gbar_D07G015620.1	GbKEA5	D07	920	99.0304	5.16	0.294	Chloroplast	24522424–24536031(+)
Gbar_D02G025040.1	GbKEA6	D02	791	86.0319	6.24	0.284	Chloroplast	67204008–67209499(+)
Gbar_A03G023150.1	GbKEA7	A03	789	86.0191	6.36	0.302	Chloroplast	104812638–104817596(+)
Gbar_D13G021490.1	GbKEA8	D13	599	64.6225	6.03	0.548	Plasma membrane	55916493–55924208(+)
Gbar_A13G021130.1	GbKEA9	A13	599	64.6596	6.03	0.557	Plasma membrane	104841961–104849912(+)
Gbar_A07G004290.1	GbKEA10	A07	575	62.205	5.91	0.661	Plasma membrane	5132112–5138369(–)
Gbar_D07G004590.1	GbKEA11	D07	575	62.1859	5.91	0.657	Plasma membrane	4798294–4804446(–)
Gbar_A08G026670.1	GbKEA12	A08	557	60.2731	5.82	0.715	Plasma membrane	119100202–119107770(–)
Gbar_D06G009660.1	GbKEA13	D06	600	65.2856	5.57	0.561	Plasma membrane	18540497–18547146(+)
Gbar_D08G027360.1	GbKEA14	D08	478	51.4877	5.35	0.753	Plasma membrane	65273584–65280951(–)
Gbar_A06G009270.1	GbKEA15	A06	563	61.3481	5.68	0.575	Plasma membrane	29086781–29093508(–)
Gbar_A07G015190.1	GbKEA16	A07	441	48.0017	6.5	0.242	Chloroplast	32524616–32547893(+)
Ghir_A08G013810.1	GhKEA1	A08	1205	130.202	5.31	0.03	Chloroplast	99333580–99342035(–)
Ghir_A12G011370.1	GhKEA2	A12	1210	130.22	5.18	0.143	Chloroplast	78653991–78663846(–)
Ghir_D08G014670.1	GhKEA3	D08	1110	119.902	5.97	0.099	Chloroplast	50279059–50287371(+)
Ghir_D12G011600.1	GhKEA4	D12	1209	129.825	5.26	0.086	Chloroplast	39774617–39784377(–)
Ghir_D02G024430.1	GhKEA5	D02	791	85.9718	6.24	0.279	Chloroplast	69322443–69327418(+)
Ghir_A03G023000.1	GhKEA6	A03	794	86.6378	6.67	0.288	Chloroplast	112523990–112529630(+)
Ghir_D13G021510.1	GhKEA7	D13	599	64.6225	6.03	0.548	Plasma membrane	59326338–59334263(+)
Ghir_A13G020690.1	GhKEA8	A13	599	64.6596	6.03	0.557	Plasma membrane	103926397–103934287(+)
Ghir_A07G004510.1	GhKEA9	A07	575	62.2781	5.91	0.653	Plasma membrane	5212492–5219763(–)
Ghir_D06G009670.1	GhKEA10	D06	596	64.794	5.57	0.554	Plasma membrane	19282756–19289573(+)
Ghir_A08G025770.1	GhKEA11	A08	588	63.8421	5.62	0.69	Plasma membrane	121887172–121893956(–)
Ghir_A06G009360.1	GhKEA12	A06	595	64.6178	5.54	0.575	Plasma membrane	31118011–31124755(–)
Ghir_D08G026630.1	GhKEA13	D08	545	58.6921	5.43	0.714	Plasma membrane	68121405–68136594(–)
Ghir_D07G004540.1	GhKEA14	D07	523	56.316	5.76	0.693	Plasma membrane	4811397–4822915(–)
Ghir_A07G015190.1	GhKEA15	A07	121	12.7251	6.24	0.908	Chloroplast	33266007–33266823(+)
Gorai.004G157200.1	GrKEA1	4	1205	130.313	5.36	0.055	Chloroplast	44542807–44551407(–)
Gorai.008G118000.1	GrKEA2	8	1209	129.88	5.26	0.084	Chloroplast	35331031–35340753(–)
Gorai.005G262000.1	GrKEA3	5	791	86.0218	6.24	0.28	Chloroplast	63648334–63653813(+)
Gorai.001G170200.1	GrKEA4	1	1011	109.902	5.85	0.085	Chloroplast	24364847–24371649(+)
Gorai.013G228100.1	GrKEA5	13	599	64.6225	6.03	0.548	Plasma membrane	54744028–54751788(+)
Gorai.004G283900.1	GrKEA6	4	573	61.7578	5.69	0.718	Plasma membrane	61514964–61521493(–)
Gorai.001G047000.1	GrKEA7	1	575	62.1759	5.91	0.648	Plasma membrane	4455718–4462880(–)
Gorai.010G101600.1	GrKEA8	10	596	64.794	5.57	0.554	Plasma membrane	18371755–18378385(+)

The results of subcellular localization prediction indicated that the proteins in KEAI were found to be located in the chloroplast (Table 3). *AtKEA1*, *-2*, *-3* in *Arabidopsis* belong to the KEAI clade were shown to be subcellular localized in chloroplasts (Aranda-Sicilia et al., 2012; Kunz et al., 2014; Sheng et al., 2014). Online tool prediction showed that the proteins in the KEAII clade were located in the plasma membrane (Table 3). However, *KEA4*, *-5*, *-6* in *Arabidopsis* belong to the KEAII clade were shown to be subcellular localized to the Golgi, trans-Golgi reticulum, and the prevacuolar compartment/multivesicular bodies (Zhu et al., 2018; Wang Y. et al., 2019). This is inconsistent with the online website prediction results, and the subcellular localization results of KEA in upland cotton require further validation.

### Phylogenetic Analysis

**FIGURE 1:**
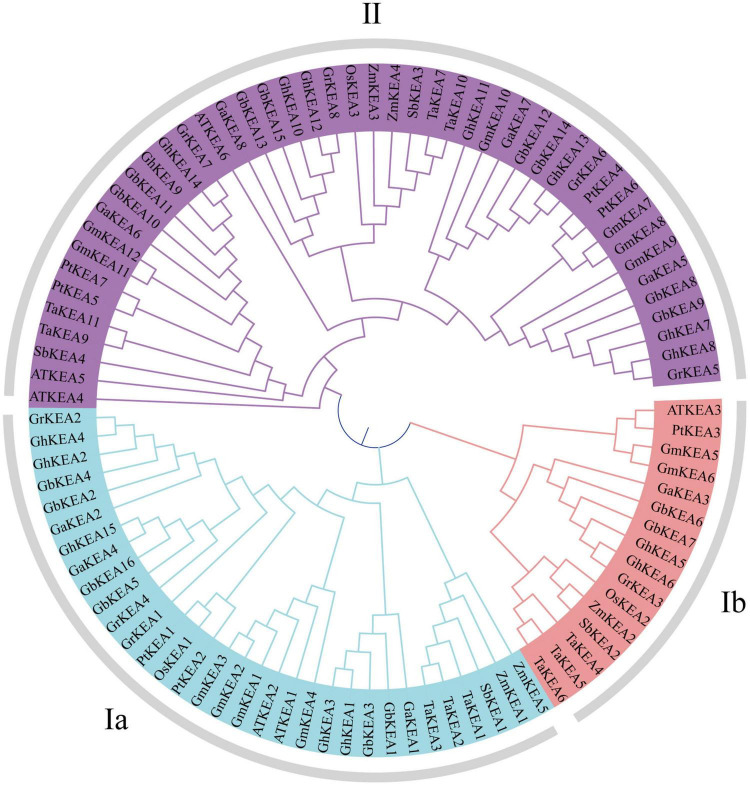
Phylogenetic relat ionship of KEA gene family. The analysis included full-length protein sequences from *G. hirsutum*, *G. raimondii*, *G. barbadense*, *G. arboreum*, *Arabidopsis thaliana*, *Oryza sativa*, *Zea mays*, *Populus trichocarpa*, *Sorghum bicolor*, *Triticum aestivum*, and *Glycine max*. Using MEGA software, 1000 bootstrap repetitive phylogenetic trees were constructed by neighbor-joining method.

Using the same method, a total of 4, 7, 7, 4,13, and 12 members were identified from *Oryza sativa*, *Zea mays*, *Populus trichocarpa*, *Sorghum bicolor*, *Triticum aestivum*, and *Glycine max*, respectively (Table 4). To examine the evolutionary relationship of KEA proteins, an unrooted phylogenetic tree was constructed using 94 KEA protein sequences from 11 species (excluding TaKEA8, TaKEA12, TaKEA13, ZmKEA6, ZmKEA7, OsKEA4 short sequences, as they did not meet the requirement of 1000 bootstrap replicates) (Figure 1). The phylogenetic tree was divided into three main categories namely KEAIa, KEAIb, and KEAII, which were consistent with the members of the AtKEA gene family (Aranda-Sicilia et al., 2012; Chanroj et al., 2012). The 11 species had distributed members in all three classifications. Among these members, the KEAII branch was the largest group, containing 46 members, while branch KEAIb was the smallest, with only 16 members. However, in the two large categories of KEAI and KEAII, the distributions of members were basically uniform. For example, 7 and 8 members of upland cotton were distributed in branch KEAI and branch KEAII, respectively. By checking the sequence characteristics, we determined that the sequence of KEAIa was the longest (except *GhKEA15* and *GbKEA16*), and the length of KEAII was the shortest.

**TABLE 4:** The KEA member ID and their names of seven species.

*Arabidopsis thaliana* ID	Name	*Oryza sativa* ID	Name 2	*Zea mays* ID	Name 3	*Populus trichocarpa* ID	Name 4	*Sorghum bicolor* ID	Name 5	*Triticum aestivum* ID	Name 6	*Glycine max* ID	Name 7
AT1G01790	ATKEA1	LOC_Os04g58620.1	OsKEA1	GRMZM2G093643_P01	ZmKEA1	Potri.002G157200.1.p	PtKEA1	Sobic.006G271800.1.p	SbKEA1	Traes_2AL_A2CBDB5F7.1	TaKEA1	Glyma.03G014000.1.p	GmKEA1
AT4G00630	ATKEA2	LOC_Os12g42300.1	OsKEA2	GRMZM2G009715_P01	ZmKEA2	Potri.014G080800.1.p	PtKEA2	Sobic.008G173800.1.p	SbKEA2	Traes_2BL_BA71DD685.1	TaKEA2	Glyma.07G073700.1.p	GmKEA2
AT4G04850	ATKEA3	LOC_Os06g36590.1	OsKEA3	GRMZM2G169114_P01	ZmKEA3	Potri.009G080800.2.p	PtKEA3	Sobic.010G168900.1.p	SbKEA3	Traes_2DL_053E73CFE.1	TaKEA3	Glyma.09G262000.1.p	GmKEA3
AT2G19600	ATKEA4	LOC_Os03g03590.1	OsKEA4	GRMZM2G171031_P01	ZmKEA4	Potri.006G230400.1.p	PtKEA4	Sobic.001G522100.1.p	SbKEA4	Traes_5DS_49CF8A4C4.1	TaKEA4	Glyma.18G230100.1.p	GmKEA4
AT5G51710	ATKEA5			GRMZM2G058948_P01	ZmKEA5	Potri.012G130500.1.p	PtKEA5			Traes_5BS_6759CB1AD.1	TaKEA5	Glyma.08G064600.1.p	GmKEA5
AT5G11800	ATKEA6			GRMZM2G040158_P01	ZmKEA6	Potri.018G054100.2.p	PtKEA6			Traes_5AS_AF90452F5.1	TaKEA6	Glyma.07G184800.1.p	GmKEA6
				GRMZM2G474078_P01	ZmKEA7	Potri.015G132400.1.p	PtKEA7			Traes_7DL_51216CD52.1	TaKEA7	Glyma.05G222500.1.p	GmKEA7
										Traes_7AL_23AA36A1B.1	TaKEA8	Glyma.08G029300.1.p	GmKEA8
										Traes_4BL_723A7E539.1	TaKEA9	Glyma.17G229700.1.p	GmKEA9
										Traes_7BL_B01B8C760.1	TaKEA10	Glyma.14G093900.1.p	GmKEA10
										Traes_5AL_91132361F.1	TaKEA11	Glyma.16G203200.1.p	GmKEA11
										Traes_4DL_59D5A8BCA.1	TaKEA12	Glyma.09G152300.1.p	GmKEA12
										Traes_4DL_806404E38.1	TaKEA13		

### Chromosome Distribution and Gene Replication Events

**FIGURE 2:**
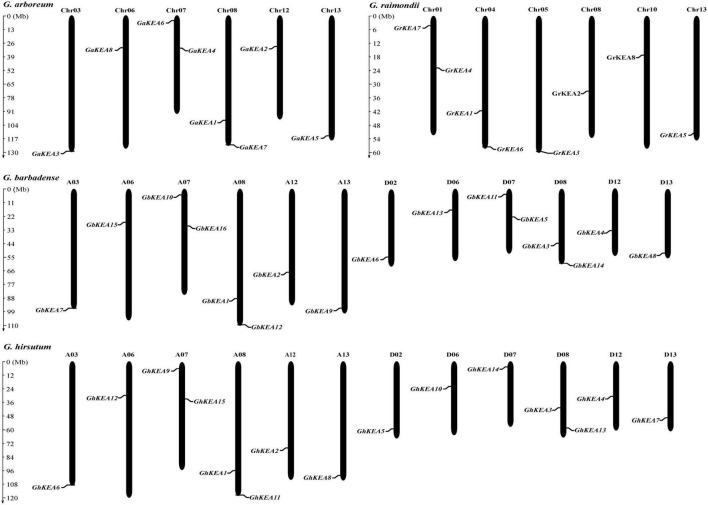
Chromosome distribution of KEAs in *G. arboreum*, *G. raimondii*, *G. hirsutum*, and *G. barbadense*. The scale stands for mega-base (Mb). The chromosome numbers are shown above each vertical line.

To determine the evolutionary relationship of KEA genes in cotton, the number and location of genes on the chromosome were analyzed (Figure 2). Each chromosome contained only one or two KEA genes. All *GaKEAs* and *GrKEAs* were distributed on 6 chromosomes, while *GhKEAs* and *GbKEAs* were distributed on six chromosomes of A_t_ subgenomes and six chromosomes of D_t_ subgenomes. Comparing the location and number of chromosomes on which the *GaKEAs* were located with the At subgenomic chromosomes on which the *GhKEAs* and the *GbKEAs* were located, we found that the location and number of genes distributed on these chromosomes of the KEA genes were basically the same. This phenomenon suggested that the distributions of KEA genes in the cotton genome were relatively conservative.

**FIGURE 3:**
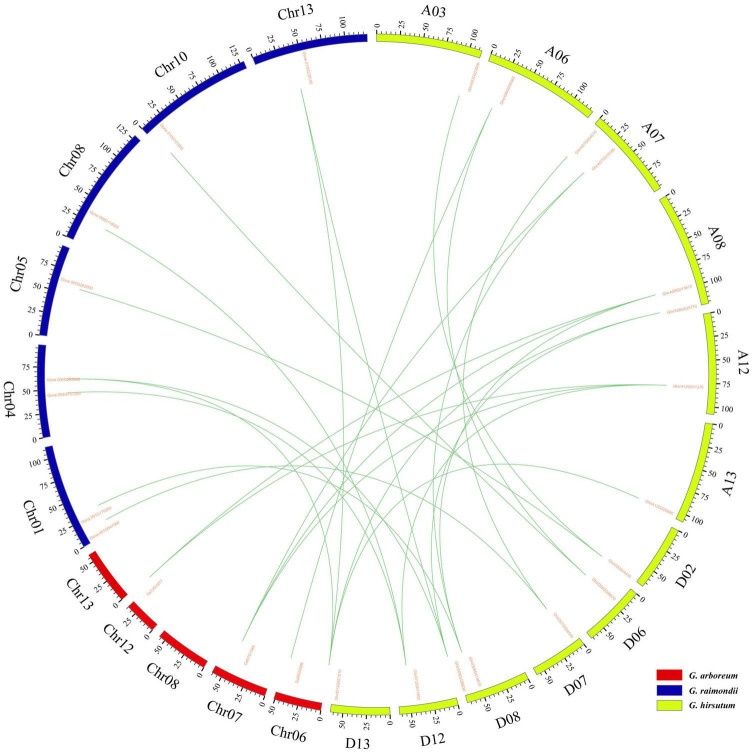
Syntenic relationships among KEA genes of two diploid (*G. arboreum*, *G. raimondii*) and one allotetraploid (*G. hirsutum*) cotton was visualized in a circos plot. The chromosomes of *G. arboreum*, *G. raimondii*, and *G. hirsutum* were shaded with red, blue, and green colors, respectively.

Gene replication events are very important for the expansion of the gene family. In general, gene replication events include tandem repeats and segmental repeats (Cannon et al., 2004; Xu et al., 2012). In this study, tandem repeat genes were defined as adjacent homologous genes on a single chromosome, and there was no more than one intermediate gene. A total of 9, 12, and 10 gene duplication pairs were identified between the A_t_ and D_t_ subgenomes of *G. hirsutum* and their corresponding ancestral A and D diploid genomes, respectively (Table 5). The data showed that all members of the KEA gene family were amplified only by segmental replication, which meant that segmental replication played a key role in the evolution of the KEA gene family. The syntenic relationships of putative KEA genes among two diploid genomes (*G. arboreum* and *G. raimondii*) and subgenomes in cultivated allotetraploid (*G. hirsutum*) were shown in Figure 3. The results showed that *GaKEAs* and *GrKEAs* were distributed among 5 and 6 chromosomes of A geneme and D genome, respectively, whereas *GhKEAs* were distributed among 6 and 6 chromosomes of A_t_ and D_t_ subgenomes, respectively. The Ka:Ks ratio can be used to judge whether the homologous gene is under positive selection pressure (Ka:Ks > 1) or purification selection pressure (Ka:Ks < 1). The results showed that the Ka:Ks ratios of GhKEA gene pairs were less than 1, indicating that KEA homologous gene pairs had undergone purifying selection during evolution and may have similar functions (Table 5).

**TABLE 5:** The Ka/Ks ratio of repetitive gene pairs between upland cotton A and D subgenomes and their corresponding ancestor An and D diploid genomes.

Gene 1	Gene 2	Ka	Ks	Ka/Ks	Duplicate
Ghir_A08G013810.1	Ghir_D08G014670.1	0.016473214	0.041483262	0.397105071	Segmental
Ghir_A12G011370.1	Ghir_D12G011600.1	0.019168907	0.044895994	0.426962539	Segmental
Ghir_A07G015180.1	Ghir_D08G014670.1	0.448822637	1.042093598	0.43069321	Segmental
Ghir_A03G023000.1	Ghir_D02G024430.1	0.010157691	0.044162866	0.23000525	Segmental
Ghir_A08G025770.1	Ghir_D13G021510.1	0.078953692	0.37203022	0.212223868	Segmental
Ghir_A13G020690.1	Ghir_D13G021510.1	0.00294515	0.030413524	0.096836869	Segmental
Ghir_A07G004510.1	Ghir_D07G004540.1	0.012239361	0.041468956	0.295145138	Segmental
Ghir_A06G009360.1	Ghir_D06G009670.1	0.005201259	0.040182906	0.129439599	Segmental
Ghir_A08G025770.1	Ghir_D08G026630.1	0.032581423	0.098093352	0.332147107	Segmental
Ghir_A08G013810.1	Ga07G1589.1	0.221236177	0.412846079	0.535880533	Segmental
Ghir_A08G013810.1	Ga08G1558.1	0.002555524	0.002299293	1.111439207	Segmental
Ghir_A08G013810.1	Ga12G1877.1	0.110829884	0.357277495	0.310206731	Segmental
Ghir_A12G011370.1	Ga07G1589.1	0.227066948	0.444887909	0.510391368	Segmental
Ghir_A12G011370.1	Ga08G1558.1	0.121462593	0.359390179	0.337968593	Segmental
Ghir_A12G011370.1	Ga12G1877.1	0.010670819	0.008999527	1.185708825	Segmental
Ghir_A13G020690.1	Ga08G2879.1	0.090571368	0.401754117	0.2254398	Segmental
Ghir_A13G020690.1	Ga13G2411.1	0	0.002297972	0	Segmental
Ghir_A08G025770.1	Ga08G2879.1	0.043530715	0.059247855	0.734722203	Segmental
Ghir_A08G025770.1	Ga13G2411.1	0.08243898	0.379636239	0.217152556	Segmental
Ghir_A06G009360.1	Ga06G0996.1	0.002966263	0.002308582	1.2848851	Segmental
Ghir_A07G015190.1	Ga07G1590.1	0.027526025	0.029670124	0.927735441	Segmental
Ghir_D08G014670.1	Gorai.001G170200.1	0.159009405	0.324757398	0.489625195	Segmental
Ghir_D12G011600.1	Gorai.004G157200.1	0.111218686	0.34816119	0.319445962	Segmental
Ghir_D12G011600.1	Gorai.008G118000.1	0.00145059	0.004628751	0.313386833	Segmental
Ghir_D02G024430.1	Gorai.005G262000.1	0.001678635	0.01382926	0.121382884	Segmental
Ghir_D13G021510.1	Gorai.004G283900.1	0.046007176	0.349474957	0.13164656	Segmental
Ghir_D13G021510.1	Gorai.013G228100.1	0	0.004594195	0	Segmental
Ghir_D06G009670.1	Gorai.010G101600.1	0	0.004611852	0	Segmental
Ghir_D08G026630.1	Gorai.004G283900.1	0.030198482	0.036981567	0.816581995	Segmental
Ghir_D08G026630.1	Gorai.013G228100.1	0.068394632	0.369738838	0.184980924	Segmental
Ghir_D07G004540.1	Gorai.001G047000.1	0.008809839	0.019963568	0.441295826	Segmental

### Analysis of Gene Structure and Conservative Motif Distribution

**FIGURE 4:**
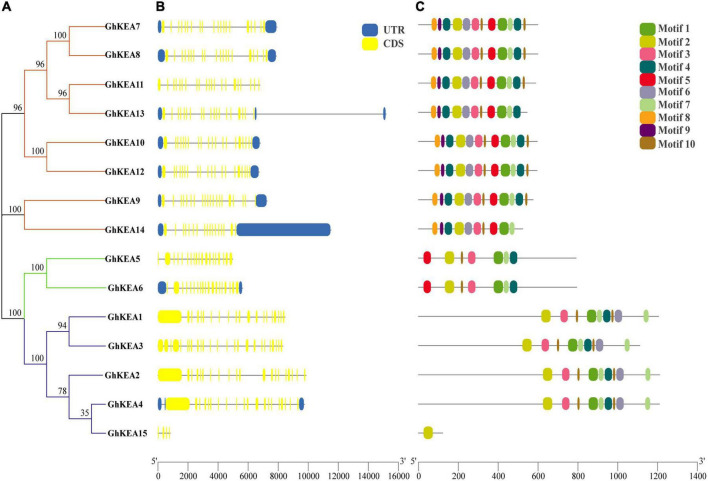
Phylogenetic relationship, exon-intron structure, and conserved motif of KEA genes in *G. hirsutum*. **(A)** The rootless tree is constructed in MEGA, and the three subfamilies are represented by different colors. **(B)** The number, length, and location of exons and introns in *GhKEAs*. Boxes and lines represent exons and introns, respectively. **(C)** Distribution of conserved motifs in the GhKEA gene family.

Phylogenetic analysis showed that the GhKEA gene family was divided into three groups, containing 8, 2, and 5 members (Figure 4A). The sequences of most members of each subfamily were similar, indicating their close evolutionary relationship. The gene length of *GhKEA15* was the shortest and that of *GhKEA13* was the longest. The exon numbers of *GhKEAs* were ranged from 4 to 20, but most of the genes contained at least 15 exons, except that *GhKEA15* contained only 4 exons (Figure 4B). The GhKEA proteins were further analyzed by the MEME program and 10 conserved motifs were identified (Figure 4C). Most GhKEA members contained multiple motifs, except GhKEA15 (1 motif), and motif 1, motif 2, motif 3, motif 4, motif 7, and motif 10 were widely distributed in these members. Motif 5 existed in the KEAIb and KEAII classes, motif 6 only did not exist in KEAIb, and motif 8 and motif 9 existed only in the KEAII class. Members of the same subfamily have similar motif characteristics, exon-intron structures and gene lengths, supporting a close evolutionary relationship.

### Analysis of *Cis*-Elements in Putative GhKEA Promoter Regions

**FIGURE 5:**
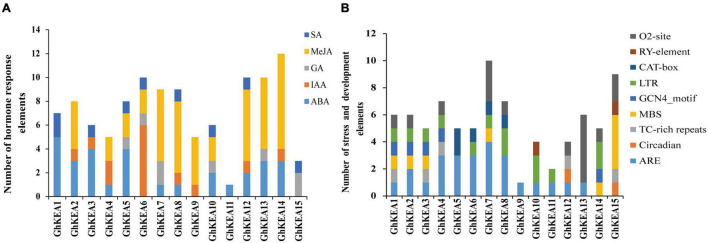
The number of plant hormone *cis*-acting elements and induced stress *cis*-acting elements in the promoter fragment of *GhKEAs*. **(A)** The sum of hormone response *cis*-acting elements in each member of *GhKEAs*. **(B)** The sum of *cis*-acting elements of stress and development in each member of *GhKEAs*.

To analyze the *cis*-elements that may be involved in the regulation of *GhKEAs*, the upstream 2000 bp sequence from the start codon (ATG) of each GhKEA gene was extracted for analysis. The *cis*-elements were classified into hormone response elements, stress response elements and plant growth and development elements (Figure 5). The hormone response elements included mainly salicylic acid (SA), methyl jasmonate (MeJA), gibberellin (GA), auxin (IAA) and abscisic acid (ABA). Most of the *GhKEAs* promoter regions contained 2–4 hormone response elements, except *GhKEA11* (1 ABA response element). Of these, *GhKEA5*, *GhKEA6*, *GhKEA8*, and *GhKEA12* contained the most hormone response elements, while *GhKEA14* contained the largest number. Among these hormone response elements located in the promoter regions of *GhKEAs*, the largest number is the MeJA response element, followed by the ABA response element (Figure 5A). Previous studies have shown that MeJA and ABA were the main plant hormone signaling molecules under stress (Mantyla et al., 1995; Pichersky and Gershenzon, 2002), so we speculated that *GhKEAs* might be involved in various stress responses of upland cotton.

The stress response elements and plant growth and development elements of the *GhKEAs* were shown in Figure 5B. *GhKEA1* contained most kinds of elements, including zein metabolism regulation elements (O2-site), low temperature response elements (LTR), endosperm expression regulatory elements (GCN4_motif), drought response elements (MBS), defense and stress elements (TC-rich repeats) and anaerobic induction elements (ARE). In addition, *GhKEA7* contained the largest number of components, while *GhKEA9* contained only one ARE element. Among the elements, the content of anaerobic inducible elements (AREs) was the highest, followed by Zein metabolic regulatory elements (O2 sites). In previous study, zein and its hydrolyzates have antioxidant activity (Diaz-Gomez et al., 2018), so we inferred that *GhKEAs* could have high antioxidant activity and antioxidant stability. These results further indicated that the KEA genes might be involved in the stress response of upland cotton.

### Organ Expression Pattern Analysis of *GhKEAs*

**FIGURE 6:**
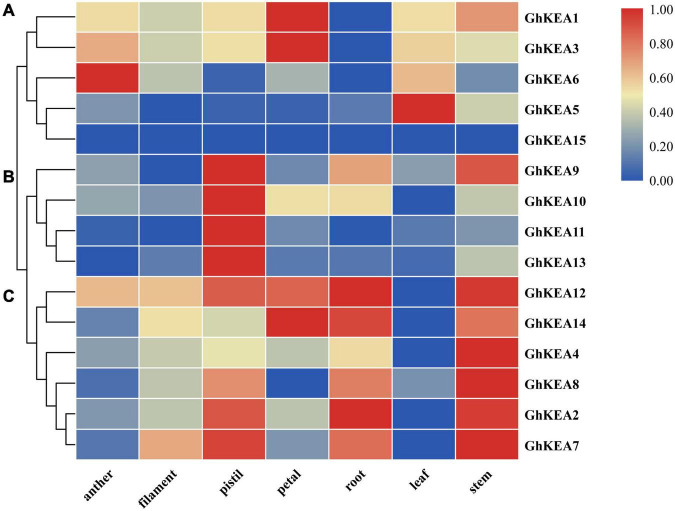
Hierarchical clustering of *GhKEAs* expression levels in ten different tissues of TM-1. The genes are displayed on the right side of each line, and the phylogenetic relationship is shown on the left **(A–C)**.

To determine the expressions of *GhKEAs* in various tissues of upland cotton, the transcription data of *GhKEAs* in different tissues (anther, filament, pistil, petal, root, leaf, and stem) in TM-1 were analyzed. As shown in Figure 6, *GhKEAs* were widely expressed in different tissues, and the same gene was highly expressed in several different tissues. The expressions of *GhKEAs* in these tissues could be divided into three groups. Group a contained *GhKEA1*, *GhKEA3*, *GhKEA5*, and *GhKEA6*, which were expressed in anthers, petals and leaves. Group b consisted of *GhKEA9*, *GhKEA10*, *GhKEA11*, and *GhKEA13*, which were highly expressed in the pistils. The last group c was composed of *GhKEA2*, *GhKEA4*, *GhKEA7*, *GhKEA8*, *GhKEA12*, and *GhKEA14*, these genes were highly expressed in the pistils, roots and stems. The multiple expression patterns indicated that the functions of *GhKEAs* had been differentiated in long-term evolution.

### *GhKEAs* Expression Patterns Under Multiple Stresses

**FIGURE 7:**
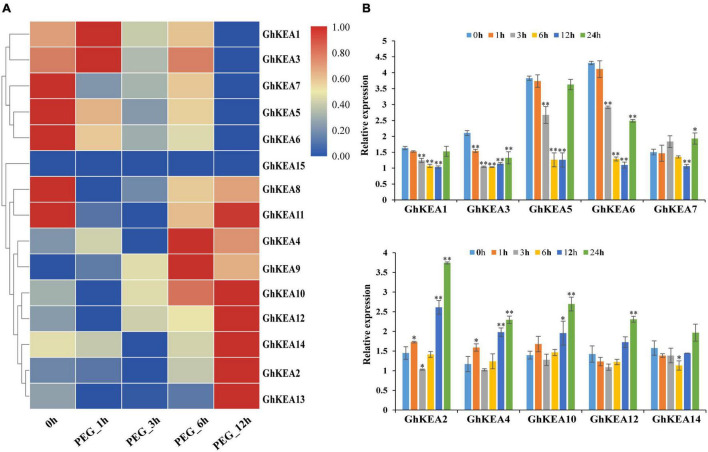
Expression profiles of *GhKEAs* under drought stress. **(A)** Hierarchical clustering of 15 *GhKEAs* expression levels under PEG treatment. **(B)** Quantificational real-time polymerase chain reaction (qRT-PCR) results of 10 *GhKEAs* under PEG treatment. 0, 1, 3, 6, 12, and 24 h represent hours after PEG treatment. The experiments were repeated three times and provided consistent results. The gene names are shown on the right. Color blocks represent the relative expression levels of *GhKEAs*. Error bars indicates SD (**p < 0.01, 0.01 < *p < 0.05, *n* = 3).

According to the analysis of *cis*-elements in the promoter region and previous studies on the KEA genes in other plants, *GhKEAs* might be involved in the stress response. To test this hypothesis, we used the available transcriptome data to analyze the expression profiles of 15 *GhKEAs* under salt and drought treatments, and further verified by qRT-PCR experiment. As shown in Figure 7A, *GhKEAs* were regulated by PEG treatments. The transcriptome data showed that the expression levels of *GhKEA2*, *GhKEA4*, *GhKEA9*, *GhKEA10*, *GhKEA12*, *GhKEA13*, and *GhKEA14* were significantly upregulated under PEG stress. However, the expression levels of *GhKEA1*, *GhKEA3*, *GhKEA5*, *GhKEA6*, and *GhKEA7* were significantly downregulated. In addition, the expression levels of *GhKEA8* and *GhKEA11* were decreased at first and then increased. Then, we selected five genes from each of the above upregulated and downregulated groups to carry out qRT-PCR experiments and further verify their response to drought stress. The data showed that *GhKEAs* expressions could be regulated by PEG treatment (Figure 7B). After PEG treatment, the expressions of *GhKEA2*, *GhKEA4*, *GhKEA10*, *GhKEA12*, and *GhKEA14* were increased, while the expressions of *GhKEA1*, *GhKEA3*, *GhKEA5*, *GhKEA6*, and *GhKEA7* were decreased, which was basically consistent with the results in the transcriptome database.

**FIGURE 8:**
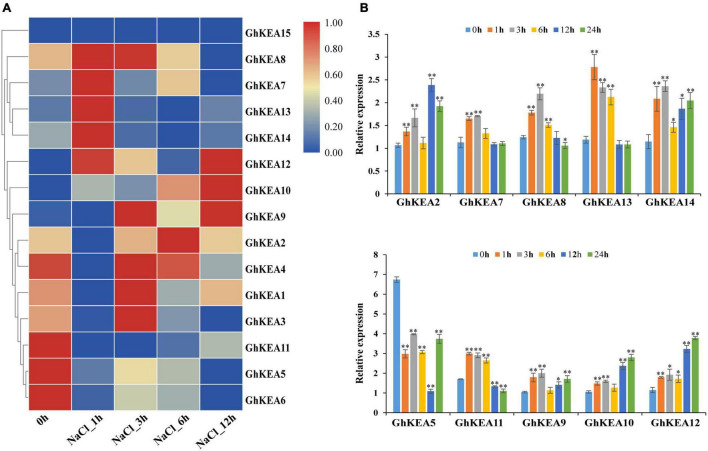
Expression profiles of *GhKEAs* under salt stress. **(A)** Hierarchical clustering of 15 *GhKEAs* expression levels under NaCl treatment. **(B)** qRT-PCR results of 10 *GhKEAs* under NaCl treatment. 0, 1, 3, 6, 12, and 24 h represent hours after NaCl treatment. The experiments were repeated three times and provided consistent results. The gene names are shown on the right. Color blocks represent the relative expression levels of *GhKEAs*. Error bars indicates SD (**p < 0.01, 0.01 < *p < 0.05, *n* = 3).

At the same time, we also analyzed the gene expression pattern under salt conditions in publicly available RNA-seq data, and the results showed that the expressions of *GhKEAs* were induced by salt stress (Figure 8A). The expressions of *GhKEA9*, *GhKEA10*, and *GhKEA12* were upregulated, while the expressions of *GhKEA5*, *GhKEA6*, and *GhKEA11* were downregulated. What’s more, the expressions of *GhKEA7*, *GhKEA8*, *GhKEA13*, and *GhKEA14* were upregulated at first and then downregulated, and reached the highest level at 1 h after treatment, while the expressions of *GhKEA1*, *GhKEA2*, *GhKEA3*, and *GhKEA4* were also upregulated and then downregulated. The difference was that the expressions of these 4 genes reached the highest level at 3–6 h. The inconsistent expression patterns of *GhKEAs* under the same stress may be due to their different promoter elements, resulting in their possible regulation by different upstream genes and thus affecting their expression patterns. We selected 10 *GhKEAs* from the above groups to further investigate the effect of salt stress on their expressions by qRT-PCR (Figure 8B). The results were basically consistent with the RNA-seq data; the expression levels of *GhKEA2*, *GhKEA7*, *GhKEA8*, *GhKEA13*, and *GhKEA14* were upregulated at first and then downregulated, *GhKEA5* and *GhKEA11* were downregulated, and *GhKEA9*, *GhKEA10*, and *GhKEA12* were upregulated.

**FIGURE 9:**
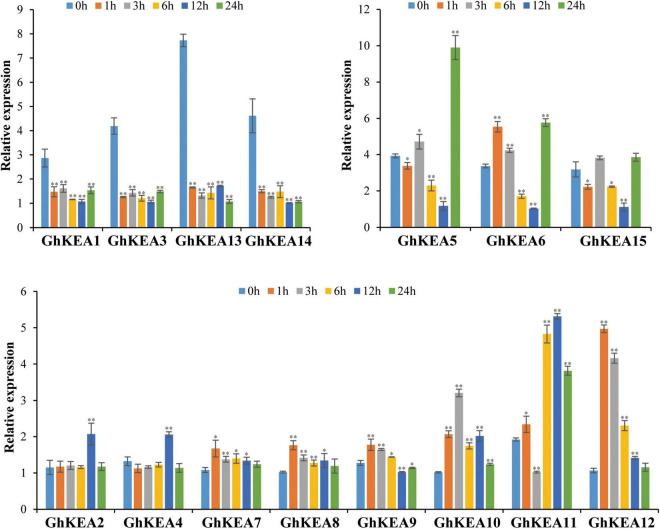
The qRT-PCR results of *GhKEAs* under low potassium treatment. 0, 1, 3, 6, 12, and 24 h represent hours after low potassium treatment. Error bars show the standard deviation of three biological repeats.

To verify the potential roles of *GhKEAs* in potassium absorption and transport, qRT-PCR experiments were used to observe whether the *GhKEAs* responded to low potassium treatment. The results are shown in Figure 9. The expressions of *GhKEAs* responded to low potassium treatment, and 15 *GhKEAs* were divided into three groups according to their expression characteristics. In group a, the expressions of *GhKEAs* were downregulated after low potassium treatment. The expressions of *GhKEA5*, *GhKEA6*, and *GhKEA15* in group b were upregulated at first, then downregulated, and then upregulated. The gene expressions in group c first increased and then decreased. Among them, the expressions of *GhKEA7*, *GhKEA8*, *GhKEA9*, and *GhKEA12* reached the highest level at 1 h after low potassium treatment, while the expressions of *GhKEA2*, *GhKEA4*, and *GhKEA11* reached the highest level at 12 h, and only *GhKEA10* reached the highest level at 3 h. These results indicated that *GhKEAs* might regulate the abiotic stress response to low potassium ions in the environment and participate in the absorption and transport of potassium ions in cotton.

### Silencing of *GhKEA4* and *GhKEA12* Compromise the Tolerance of Cotton to Salt Stress

**FIGURE 10:**
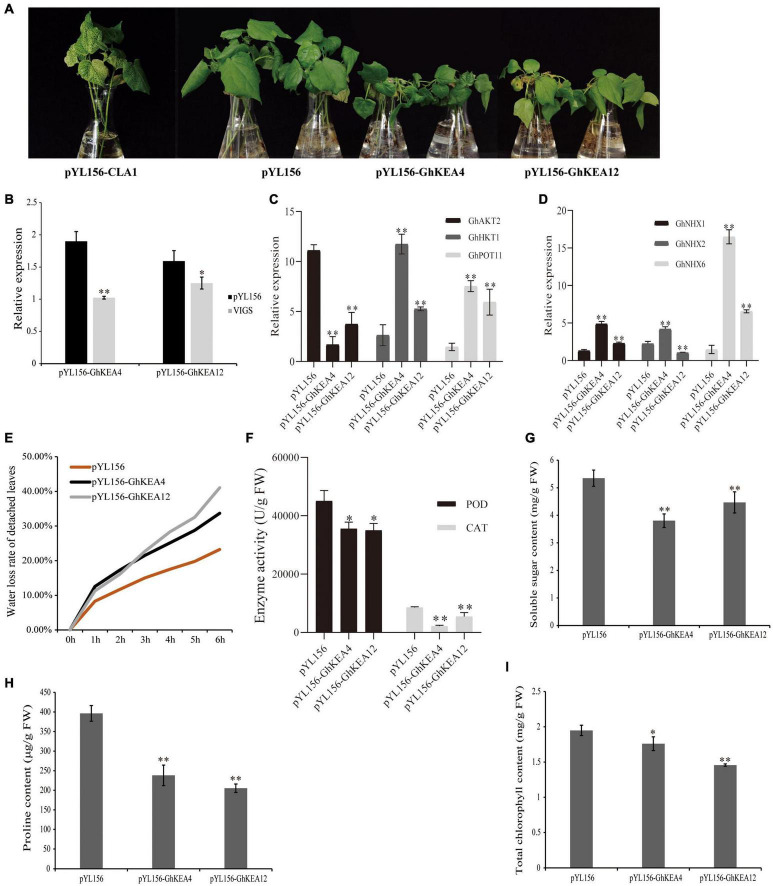
Silencing *GhKEA4* and Silencing *GhKEA12* in cotton can destroy the tolerance of cotton to salt stress. **(A)** The phenotypes of positive control, empty control, and virus-induced gene silencing (VIGS) plants under salt treatment. **(B)** The expression levels of *GhKEA4* and *GhKEA12* in pYL156 and VIGS plants leaves. **(C,D)** Relative expression levels of K and Na transport related genes in pYL156 and VIGS plants leaves. **(E)** The water loss rate of isolated leaves of control plants and VIGS plants leaves. **(F)** The activities of CAT and POD in empty control and VIGS plants leaves after salt treatment. **(G–I)** After salt treatment, the contents of soluble sugar, proline and chlorophyll in empty control and VIGS plants leaves. Error bars indicate SD (**p < 0.01, 0.01 < *p < 0.05, *n* = 3).

Analysis of the promoter regions of *GhKEAs* revealed that the promoter regions of both *GhKEA4* and *GhKEA12* contain ABA and MeJA hormone response elements as well as antioxidant response elements ARE. *GhKEA12* was significantly differentially expressed under low potassium stress and significantly up-regulated by drought and salt stress. The expression of *GhKEA4* was significantly up-regulated under drought stress, while it also responded to low potassium and high salt stress. Then, *GhKEA4* and *GhKEA12* were selected for the VIGS experiments and salt treatments. The albino phenotype on the pYL156-*CLA1* cotton plant ensured the success of the VIGS experiment (Figure 10A). As shown in Figure 10B, the expressions of the *GhKEA4* and *GhKEA12* in the corresponding VIGS plants leaves were significantly lower than the expressions in the pYL156 empty vector plants, indicating that the genes had been silenced successfully. Then these plants were treated with salt to observe the phenotype. Figure 10A showed that after salt stress, cotton plants silenced for *GhKEA4* and *GhKEA12* showed obvious wilting compared with control cotton plants. Subsequently, the leaves of these plants were treated with drought *in vitro*, to calculate the water loss rate of detached leaves. The results showed that the water loss rates of cotton leaves were significantly higher than that of control plants after silencing *GhKEA4* and *GhKEA12* genes, indicating that the water holding capacity of leaves decreased after silencing the target gene (Figure 10E). In order to further investigate the effects of salt stress on the physiological and biochemical characteristics of plants, the chlorophyll content, proline content, soluble sugar content, peroxidase (POD) activity and catalase (CAT) activity in VIGS cotton leaves were measured after salt stress. The results showed that after salt treatment, CAT and POD in control plants were extremely significantly and significantly higher than those in plants silenced for the target gene (Figure 10F). Furthermore, the soluble sugar, proline and total chlorophyll contents in the leaves of control plants were significantly higher than those of plants silenced for the target gene (Figures 10G–I). These results indicated that the salt tolerance of cotton plants decreased after silencing *GhKEA4* and silencing *GhKEA12*.

In addition, the results of the expression levels of related genes encoding K and Na transporters showed that *GhAKT2* was down-regulated, *GhHKT1*, *GhPOT11*, *GhNHX1*, and *GhNHX6* were up-regulated in plants silenced for the target gene, while *GhNHX2* was up-regulated in plants silenced for *GhKEA4* and down-regulated in plants silenced for *GhKEA12* (Figures 10C,D).

### K^+^ and Na^+^ Contents in Virus-Induced Gene Silencing Cotton Plants Under High Salt, High Potassium, and Low Potassium Stress

**FIGURE 11:**
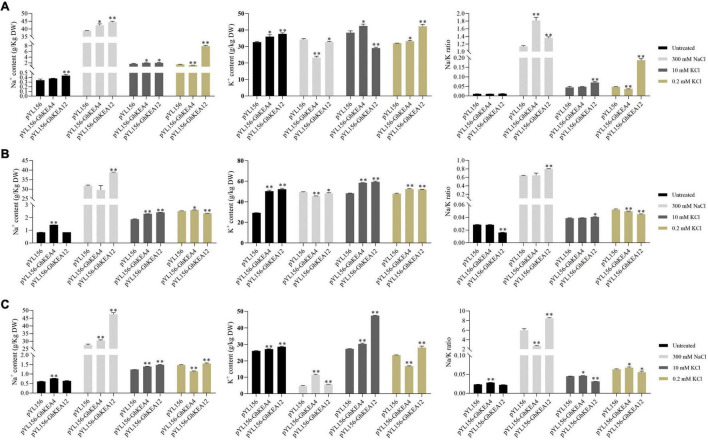
Changes in Na^+^, K^+^ content and Na^+^/K^+^ ratio in plants injected with empty vector and in plants silenced for *GhKEA4* and silenced for *GhKEA12* under high salt, high and low potassium stress. **(A)** Leaf. **(B)** Stem. **(C)** Root. Error bars indicate SD (^**^p < 0.01, 0.01 < *p < 0.05, *n* = 3).

In order to preliminarily characterize the potassium ion transport activity of KEA gene, the K^+^ and Na^+^ content in cotton silenced for *GhKEA4* and *GhKEA12* before and after high salt, high potassium, and low potassium treatments were measured. The results showed that under control conditions (untreated), after silencing *GhKEA4* in cotton plants, the Na^+^ content in stems and roots was extremely significantly higher than that in empty vector control plants, the K^+^ content in the entire plant was significantly increased, but only the Na^+^/K^+^ ratio in roots was extremely significantly increased (Figures 11B,C). After silencing the *GhKEA12* gene in cotton plants, the Na^+^ content in leaves was extremely significantly higher than that in empty vector control plants, the K^+^ content in the entire plant was extremely significantly increased, and only the Na^+^/K^+^ ratio in stems was extremely significantly decreased (Figure 11A). The results showed that the Na^+^/K^+^ balance could be basically maintained after silencing *GhKEA4* and *GhKEA12* genes.

Under salt stress, after silencing the *GhKEA4* gene in cotton plants, the Na^+^ content in leaves and roots was significantly higher than that in unloaded control plants, the K^+^ content was significantly decreased in leaves and stems and significantly increased in roots, and the Na^+^/K^+^ ratio was significantly increased in leaves and significantly decreased in roots (Figure 11), indicating that silencing the *GhKEA4* gene decreased the potassium ion transport activity from the lower ground to the shoot of plants under salt stress, resulting in a higher Na^+^/K^+^ ratio in leaves. After silencing *GhKEA12*, the Na^+^ content in the plants was extremely significantly increased, the K^+^ content was extremely significantly increased in the roots, significantly decreased in the leaves and stems, and the Na^+^/K^+^ ratio in the plants was extremely significantly increased (Figure 11), indicating that the potassium transport activity was decreased and the sodium transport activity was increased in the plants silenced *GhKEA12*, resulting in the plants with a higher Na^+^/K^+^ ratio.

Under high potassium treatment, the contents of Na^+^ and K^+^ in plants silenced with *GhKEA4* increased significantly, but their Na/K ratio did not change significantly (Figure 11), indicating that cotton silenced with *GhKEA4* could still maintain a more stable Na^+^ and K^+^ balance. Additionally, Na^+^/K^+^ ratio was significantly increased in leaves and stems and significantly decreased in roots in *GhKEA12*-silenced plants, indicating that potassium ion transport activity from roots to leaves was reduced in plants with *GhKEA12* gene silencing.

Under low potassium stress, the Na^+^ content in leaves and roots of plants silenced for the *GhKEA4* was extremely significantly decreased and significantly increased in stems. K^+^ content was significantly increased in leaves and stems and extremely significantly decreased in roots. Na^+^/K^+^ ratio was extremely significantly decreased in leaves and stems and significantly increased in roots (Figure 11), indicating that silencing of *GhKEA4* gene increased the activity of plants to transport potassium to shoots under low potassium conditions; K^+^ content in plants silenced with *GhKEA12* was extremely significantly increased, the Na^+^ content in leaves and roots was extremely significantly increased in stems, Na^+^/K^+^ was extremely significantly increased in leaves, and significantly decreased in stems and roots, indicating that silencing with *GhKEA12* gene improved the ability of plants to absorb potassium, but accumulated more Na^+^ in leaves, resulting in higher Na^+^/K^+^ in leaves.

## Discussion

The *AtKEAs* are homologous to EcKefB and EcKefC of *Escherichia coli* (Chanroj et al., 2012). When EcKefB/EcKefC binds to its helper proteins EcKefF and glutathione, the conformation of the KTN domain changes, which turns on the potassium ion transport switch of EcKefB/EcKefC (Miller et al., 2000; Roosild et al., 2002, 2009, 2010). Some studies have shown that the expressions of *AtKEA1*, *AtKEA3*, and *AtKEA4* were enhanced under low potassium stress, and the expressions of *AtKEA2* and *AtKEA5* were enhanced under sorbitol and abscisic acid treatment (Aranda-Sicilia et al., 2012; Kunz et al., 2014; Zhu et al., 2018). The CPA family in some plants has been identified and verified (Maser et al., 2001; Chanroj et al., 2012; Ye et al., 2013; Zhou et al., 2016; Sharma et al., 2020), but there are few studies to identify the CPA family in cotton, especially the KEA family, which is a subfamily of the CPA family. In the current study, we identified the members of the KEA family in cotton by sequence similarity, and then carried out a comprehensive bioinformatics analysis of the KEA gene in cotton. The comprehensive analysis of the characteristics of the cotton KEA gene family will provide a basis for further research.

### Evolution and Characterization of the K^+^ Efflux Antiporter Gene Family in Cotton Species

Genome-wide doubling events occurring in the process of plant evolution have had a lasting and far-reaching impact on plants, and some plants have even experienced whole-genome doubling events repeatedly (Tuskan et al., 2006). Approximately 130 million years ago, the common ancestor of dicotyledons experienced a genome-wide triploid event (Jaillon et al., 2007). Then, cotton independently experienced a genome-wide pentaploid event (Paterson et al., 2012; Wang et al., 2016). Allotetraploid upland cotton enlarged the number of genes after multiple replication events. In this study, 8, 8, 15, and 16 KEA genes were identified in *G. raimondii*, *G. arboreum*, *G. hirsutum*, and *G. barbadense*, respectively. The number of KEA genes in tetraploid cotton is approximately the sum of KEA genes in *G. arboreum* and *G. raimondii*. The unbalanced distribution of KEA genes on each chromosome number proved the existence of genetic variation in the process of evolution (Paterson et al., 2012; Zhu and Li, 2013; Yu et al., 2014). Based on the classification of the KEA gene family in *Arabidopsis* (Maser et al., 2001), all putative KEA genes in this study can be divided into three subfamilies. The lineal homologous genes of monocotyledons tend to form lineal homologous gene pairs at the end of the branches of phylogenetic trees, while the *KEA* genes of dicotyledons tend to be clustered together, possibly due to the different functions of KEA proteins in monocotyledons and dicotyledons (Li et al., 2016). Furthermore, the gene members of each subfamily not only have similar gene structure, sequence length and motif structure, but also have the same results of subcellular location prediction. These results suggested that the members of the KEA gene family may show relatively conservative functions in the growth of upland cotton, especially those of the same subfamily (Palusa et al., 2007). The similarities and differences in the gene structure, domain and motif of *GhKEAs* may be related to the long evolutionary history and gene replication of cotton (He and Zhang, 2005). In the process of gene family evolution, tandem replication and segmented replication contributed to the emergence of gene families to a certain extent. We found that *GhKEAs* could be amplified only by segmental replication, indicating that segmental replication played a key role in the evolution of the GhKEA gene family. Collinear analysis showed that most of the KEA homologous gene pairs between the A_t_ and D_t_ subgenomes of *G. hirsutum* and their corresponding A and D diploid genomes were located in the collinear region. Based on these results, we speculated that whole genome replication was the main driving force for the expansion of the KEA gene from diploid to allotetraploid.

### *GhKEAs* May Play an Important Role in Facilitating K^+^ Homeostasis

Maintaining the homeostasis of intracellular ions is not only the basic cellular activity needed for plant growth, but also the basis for regulating plant growth and development and coping with environmental stress (Yang et al., 2019). Previous studies have shown that *AtKEA1*, *-3*, and *-4* are induced by low potassium stress (Zheng et al., 2013) and that the AtKEA gene family plays a key role in K^+^ homeostasis and osmoregulation (Zheng et al., 2013; Kunz et al., 2014; Zhu et al., 2018). In this report, the expression characteristics of *GhKEAs* under low potassium stress were characterized for the first time, and most of the *GhKEAs* were induced by low potassium stress. For example, *GhKEA12*, as an ortholog of *AtKEA4*, showed significant changes in its expression levels under low potassium stress. Many studies have demonstrated that the KEA family with Na^+^/H^+^ exchanger domains and NAD-binding (KTN) domains participates in the absorption and transport of potassium ions (Qiu et al., 2003; Bölter et al., 2020). Therefore, to preliminarily explore the involvement of *GhKEAs* in K^+^ transport, the contents of K^+^ and Na^+^ in the leaves of silenced *GhKEA4* cotton plants and silenced *GhKEA12* cotton plants were examined. In addition, silencing *GhKEA4* and *GhKEA12* decreased the activity of transporting potassium ions in leaves under high salt condition, resulting in higher Na/K ratio in leaves. Silencing *GhKEA12* inhibited K^+^ transport activity and increased Na^+^ content in cotton leaves under high potassium stress. Silencing *GhKEA12* increased K^+^ transport activity and Na^+^ absorption capacity under low potassium stress. However, the effect of silencing *GhKEA4* gene on K^+^ transport activity in plant leaves was very low under high and low potassium stresses. *GhKEA4* is an ortholog of *AtKEA2*, and it has been shown that *AtKEA2* is involved in K^+^ homeostasis in chloroplasts or plastids (Aranda-Sicilia et al., 2012), which is consistent with our results. Alternatively, it has also been shown that *AtKEA2* has an important function in maintaining local osmotic pressure, ionic and pH homeostasis, and the formation of thylakoid membranes (Kunz et al., 2014; Ali, 2016; Aranda-Sicilia et al., 2016). While *GhKEA12* is an ortholog of *AtKEA6*, *AtKEA6* likewise plays a role in maintaining ion homeostasis (Zhu et al., 2018), as indicated by the K^+^ uptake system (Tsujii et al., 2019). Overall, our results demonstrated that the *GhKEAs* were involved in regulating the dynamic balance of intracellular K^+^ during the growth and development of cotton.

It has been shown that *AKT1* is involved in K^+^ uptake in the micromolar concentration range (Spalding et al., 1999); *HKT1* and *KUP7* have an important role in regulating K homeostasis in plants (Han et al., 2016; Wang et al., 2018); and Na^+^ and H^+^ exchange rates are significantly increased in vacuoles of plants overexpressing the *AtNHX1* gene (Apse et al., 1999). We found significant changes in the expression levels of these genes in cotton silenced for *GhKEA4* or *GhKEA12* genes. These results suggest that there may be a “genetic compensation” mechanism for potassium ion absorption and transport in cotton.

### GhKEAs Regulate Cotton Response to Salt Stress

We analyzed the *cis*-acting elements in the promoter region of the upland cotton KEA gene family and found a variety of stress-responsive *cis*-acting elements such as TC-rich repeats and MBS. Studies have shown that ABA and MeJA played an important role in regulating plant stress responses (Reyes and Chua, 2007; Tavallali and Karimi, 2019). ABA response elements (ABREs) and MeJA response elements (TGACG-elements and CGTCA-motifs) were obtained in the *cis*-acting elements of the GhKEA gene family. Moreover, qRT-PCR results showed that the GhKEA gene family responded positively to stress in the early or late stages of salt and drought treatments. When plants are subjected to salt stress, a large number of reactive oxygen species accumulate, leading to lipid peroxidation and interfering with the normal physiological process (Jithesh et al., 2006). POD and CAT are important antioxidant enzymes for scavenging reactive oxygen species in plants (Harb et al., 2010). At the same time, proline and soluble sugar, as important substances regulating plant cell osmotic potential, promote the scavenging of intracellular reactive oxygen species to some extent (Ullah et al., 2018). In this study, *GhKEA4* and *GhKEA12*, which were sensitive to salt treatment, were selected for VIGS experiments. The results showed that the water loss rate of detached leaves, the contents of proline and soluble sugar, and the activities of POD and CAT in VIGS plants were lower than those in the blank control to varying degrees. *AtKEA2* and *AtKEA6*, paralogs of *GhKEA4* and *GhKEA12*, have been reported to confer tolerance to high Na^+^ stress in *Arabidopsis* (Aranda-Sicilia et al., 2012; Wang Y. et al., 2019). It has been reported that salt stress-induced production of ROS can promote K^+^ entry into the cytoplasm, thereby reducing the Na^+^/K^+^ ratio (Ma et al., 2012). We speculated that excessive accumulation of ROS in plants under salt stress may further promote *GhKEAs* to transport K^+^, reduce the Na^+^/K^+^ ratio, and then promote the accumulation of osmoregulatory substances to improve tolerance to abiotic stress, and the conclusion needs further verification.

## Conclusion

Under salt stress, plants accumulate a large amount of Na^+^ and inhibit the absorption of K^+^, resulting in an imbalance in the ion dynamic balance. Ion transporters can maintain ion homeostasis in plant inner membrane systems. In the present study, K^+^ efflux transporters were identified in *Gossypium* spp. Then, the distribution, sequence structure and expression pattern of the KEA gene family in cotton were analyzed in detail at the whole genome level, as well as its potential function in cotton growth and development and response to abiotic stress. In addition, VIGS experiments were used to be verified that the *GhKEAs* could maintain a relatively stable Na/K ratio in upland cotton under high salt, high potassium and low potassium stresses, as well as play an important function in salt stress. The comprehensive analysis of KEA genes in this study lays a foundation for future functional research on cotton KEA genes.

## Data Availability Statement

Publicly available datasets were analyzed in this study. This data can be found at the National Center for Biotechnology Information Search database (https://www.ncbi.nlm.nih.gov/) under accession numbers PRJNA382310, PRJNA171262, PRJNA433615, PRJNA10719, PRJNA448171, PRJNA374837, ACUP00000000, and PRJNA248163.

## Author Contributions

YL: conceptualization, methodology, data curation, software, original draft, and writing – review and editing. ZF: methodology and software. HWe, SC, and PH: methodology. HWa: conceptualization, supervision, and writing – review and editing. SY: conceptualization and supervision. All authors contributed to the article and approved the submitted version.

## Conflict of Interest

The authors declare that the research was conducted in the absence of any commercial or financial relationships that could be construed as a potential conflict of interest.

## Publisher’s Note

All claims expressed in this article are solely those of the authors and do not necessarily represent those of their affiliated organizations, or those of the publisher, the editors and the reviewers. Any product that may be evaluated in this article, or claim that may be made by its manufacturer, is not guaranteed or endorsed by the publisher.

## Footnotes

1
https://www.cottongen.org/

2
https://phytozome-next.jgi.doe.gov/

3
https://www.ncbi.nlm.nih.gov/Structure/bwrpsb/bwrpsb.cgi

4
http://smart.embl-heidelberg.de/

5
https://web.expasy.org/protparam/

6
http://linux1.softberry.com/berry.phtml?topic=protcomppl&group=programs&subgroup=proloc

7
http://gsds.gao-lab.org/

8
http://bioinformatics.psb.ugent.be/webtools/plantcare/html/
